# Time to Recovery From Severe Acute Malnutrition to Normal Nutritional Status and Its Predictors Among Children Aged 6–59 Months in North‐East Ethiopia

**DOI:** 10.1111/mcn.13808

**Published:** 2025-02-16

**Authors:** Temesgen Gebeyehu Wondmeneh, Amarech Giruma

**Affiliations:** ^1^ Department of Public Health College of Medical and Health Science Samara University North‐East Afar Ethiopia

## Abstract

Malnutrition is a major disease burden in developing countries, particularly in recurrently drought‐affected areas. Despite the Ethiopian government's initiatives to set up stabilization centers in different hospitals to tackle severe acute malnutrition, there is limited data on the time to recover from severe acute malnutrition and its determinants among under‐five children in northeast Ethiopia. The objective of the study is to determine time to recovery of under‐five children from severe acute malnutrition to normal nutritional status and its predictors in northeast Ethiopia. A facility‐based retrospective record review was carried out from March 1–20, 2023. The tools for the data extraction format were adapted from the national guidelines for the management protocol for severe acute malnutrition. The Kaplan–Meier survival curve was used to compare different categorical variables. The time‐varying covariate Cox‐proportional hazards regression model was fitted due to the violation of the Cox proportional hazard assumption (*p* = 0.007). A *p*‐value < 0.05 was a cutoff point to declare statistical significance. In the final analysis, a total of 372 children aged 6–59 months with severe acute malnutrition were included, 58.1% of whom were recovered. The incidence rate of recovery from severe acute malnutrition was 4.43 per 100 child days. Children living in rural areas (AHR = 0.7, 95% CI: 0.5–0.94) and those without F‐100 supplement (AHR = 0.85, 95% CI: 0.79–0.91) had a lower recovery rate from severe acute malnutrition. Children lacking IV antibiotics (AHR = 1.4, 95% CI: 1.03–2.0) and those HIV‐free (AHR = 1.76, 95% CI: 1.1–3.3) were more likely to recover from severe acute malnutrition. The percentage of recovery in the study area was found to be lower than the sphere standard. F‐100 supplements should be mandatory to improve and speed up the recovery rate. Special attention should be given to children from rural areas, those receiving IV antibiotics, and those living with HIV/AIDS.

## Summary

- The percentage of recovery in the study area was found to be lower than the sphere standard.
- F‐100 supplements should be mandatory to improve and speed up the recovery rate.
- Children living in rural areas and those with IV antibiotics and HIV/AIDS should be closely monitored and receive additional medical care to improve the recovery rate.

**Abbreviations**
AHR: adjusted hazard ratio
CI: confidence interval
Fig: figure
HIV/AIDS: human immunodeficiency virus/acquired immune deficiency syndrome
IV: intravenous
MUAC: mid‐upper arm circumference
S: supplementary
SPSS: social science statistical package
TB: tuberculosis
WASH: water and sanitation hygiene
WHO: World Health Organization

## 1 Introduction

Malnutrition is the main cause of disease worldwide and is an important global problem with substantial social and economic impacts (John‐Joy John‐Joy Owolade et al. 2022). By 2030, the zero hunger targets will mostly be missed if current trends continue (UN 2023). Children under 5 years of age are the most vulnerable to malnutrition (Chawla et al. 2020). In children aged 6–59 months, severe acute malnutrition is detected based on anthropometric measurements and the presence of nutritional edema. A very low mid‐upper arm circumference (MUAC < 115 mm) or a weight‐for‐height z score below −3 of the median value from the WHO 2006 reference data are indicators of severe wasting (Otiti and Allen 2021). Children with severe acute malnutrition experience significant developmental delays after being admitted to the hospital (van den Heuvel et al. 2017). The mortality rate for children with moderate to severe acute malnutrition is three to nine times higher than that for children who are adequately nourished (Ghosh‐Jerath et al. 2017). According to the 2020 WHO report, 47.0 million children under the age of 5 were wasted, 14.3 million were severely wasted, and over a third of them lived in Africa (Govender et al. 2021). In 2022, an estimated 45 million children under the age of five (6.8%) were affected by wasting (WHO 2023). According to Sphere standards, the minimum acceptable recovery rate from severe acute malnutrition is greater than 75% (Sphere 2018). In sub‐Saharan Africa, the overall rate of recovery from severe acute malnutrition in children under the age of 5 years was 71.2% (Desyibelew et al. 2020). Studies conducted in Ethiopia between 2019 and 2020 showed that the recovery rate of children from severe acute malnutrition ranged from 70% to 72% (Bitew, Alebel, Worku 2020; Yazew et al. 2019). The proportion of recovery, incidence rate, and median time of recovery in the district‐level studies of Ethiopia are shown in Table 1.

**Table 1:** Shows the proportion of recovery, incidence rate, and median time of recovery in the district‐level studies of Ethiopia.

District study site	Percentage of recovery	Incidence rate of recovery	Median time of recovery	Study period	References
Oromia region	68.72%	3.35/100‐person day	21 days	2017–2021	Eyi et al. (2022)
Amhara region	62.13%	—	16 days	2012–2016	Baraki et al. (2020)
Dire Dawa	79.8%	—	8.7 weeks	2013–2016	Atnafe et al. (2019)
Southwest Ethiopia	54.4%	—	49 days	2018–2019	Wondie et al. (2022)
Southern Ethiopia	70.4%	—	7 weeks	2017–2018	Simachew et al. (2020)
Southern Ethiopia	69.3%	3.8/100 person‐days	17 days	2015–2017	Fikrie et al. (2019)
Addis Ababa	81.3%	5.29/100 person‐day	15 days	2016–2018	Adimasu et al. (2020)
Northwest Ethiopia	65.3%	1.94/100 person‐day	38.5 days	2017	Mamo et al. (2019)
Tigray region	75.9%	54/1000 person‐days	16 days	2020	Kidane et al. (2023)
Metekel district	65.8%	5.3/100 person‐day	14 days	2013–2017	Wondim et al. (2020)
Asosa district	65.4%	5.28/100 child‐days	15 days	2015–2019	F. K. Bizuneh et al. (2022)
Jimma district	73.1%	4.06/100 person‐days	19 days	2015–2017	Hussen Kabthymer et al. (2020)

*Note:* Dash (—) indicated not reported.

The recovery rate can be affected by a number of factors, such as socio‐demographic, economic, environmental, clinical, and improper implementation of the treatment protocols (Bitew, Alebel, Worku, & Alemu 2020). Studies in Ethiopia revealed that children with severe acute malnutrition who had HIV, TB, malaria, and pneumonia had poor recovery when compared to children who did not have these conditions (Aye et al. 2023; Baraki et al. 2020; Oumer et al. 2021). Children with HIV plus severe acute malnutrition had higher levels of inflammatory mediators than children with severe acute malnutrition alone (Sturgeon et al. 2023). Children who had diarrhea and vomiting had a lower recovery rate from severe acute malnutrition than children who did not have diarrhea and vomiting (Husen et al. 2022; Wondie et al. 2022), respectively. In a study in Ethiopia, the average weight gain and recovery time of children treated with ready‐to‐use therapeutic food (RUTF) significantly improved compared to children without RUTF (Abebe et al. 2023). Other studies conducted in Ethiopia revealed that children on Formula 100 (F‐100) milk supplements (F. Bizuneh et al. 2021; Fikrie et al. 2019), lacking nasogastric intubation (F. Bizuneh et al. 2021; Oumer et al. 2021) and taking amoxicillin (Husen et al. 2022; Hussen Kabthymer et al. 2020), had a better recovery rate than children without F‐100 milk supplements, those with nasogastric intubation, and those who did not take amoxicillin, respectively. A study in Chad showed that the provision of a household WASH package (Altmann et al. 2018) improved the recovery rate of children from severe acute malnutrition by 10.5%. Household food insecurity is a contributing factor for poor recovery of children from severe acute malnutrition (Kabalo 2018). Early marriage increased the risk of children's malnutrition (Paul et al. 2019). Although the prevalence of malnutrition among under‐five children in the Afar region as reported in 2019 was high (wasting, stunting, and underweight were 16.2%, 43.1%, and 24.8%, respectively) (Gebre et al. 2019), there is no data regarding the recovery rate and its predictors for time to recovery. Therefore, this study investigated the recovery rate of children from severe acute malnutrition and its determinants since this research is a health need or priority of the Afar community. The findings of this study can be used to inform public health initiatives and improve service management of severe acute malnutrition by assessing how children respond to RUTF.

## 2 Methodology

### 2.1 Study Area

The study was conducted at Dubti and Aysaita public Hospitals, located in Awsi Rasu Zone, Afar Region, at the northern tip of the Great East African Rift Valley in the northeast part of Ethiopia. The Afar region's climate is hot, dry, and affected by recurrent drought. The availability of food is often scarce each year. The majority of Afar people are pastoralists. Aid organizations provide support in the region under the Productive Safety Net Program (PSNP) due to the vulnerability of people to flooding and drought (Wondmeneh 2022). The food security situation in the Afar region has gotten worse since conflict began to pick up in early November 2020. Sixty percent of the Afar people are experiencing food insecurity at crisis levels (FAO 2021). Pastoralists in the Afar region had received poor‐quality water supplies (Libey et al. 2022). Early marriage is the most common practice in Afar (Alem et al. 2020; Dessalegn et al. 2020). Pre‐lacteal feeding practice, which is one of the causes of malnutrition, is widely practiced in the region due to the cultural perspective of the Afar community in the region (Wondmeneh 2023). There is a scarcity of health centers and health care practitioners in the Afar region (Addis and Gebeyehu Wondmeneh 2023). According to the 2007 census, Awsi Rasu Zone has an estimated total population of 551,173. The Dubti hospital is the only largest referral hospital for the Afar region, where patients from all over the pastoral areas of Afar receive referral services, while the Aysaita hospital is also the second largest district hospital in the region and serves the Afar people. These two hospitals provide a wide range of services for the Afar people who come from different places in the region. The number of beds in Dubti Hospital and Aysaita Hospital is 129 and 68, respectively. Using these two hospitals helped to ensure the accurate capture of cases from all geographical areas of Afar (to cover all populations, even those who are marginalized), contributing to the sample's representativeness. Stabilization centers are unique in that they provide specialized care for children with severe acute malnutrition and medical complications. These two hospitals served as stabilizing centers for children who required admission due to severe acute malnutrition and who were referred from all over the Afar region.

### 2.2 Study Design and Period

A facility‐based retrospective cohort study design was conducted based on a record review of children aged 6–59 months with severe acute malnutrition registered in stabilization centers from March 1 to 20, 2023, at Dubti Hospital and Aysaita Hospital.

### 2.3 Study Population

The study populations were all children aged 6–59 months with severe acute malnutrition who were admitted from January 1, 2022 to December 31, 2022, at Dubti Hospital and Aysaita Hospital. Children with incomplete medical records on outcome variables, baseline socio‐demographic data, and anthropometric measurements were excluded. Children with an unrecorded admission or discharge date were excluded. Furthermore, children who were admitted to these two hospitals more than once due to severe acute malnutrition would only be included in the first episode because the recovery rate or therapeutic response of children with relapse severe acute malnutrition may be different from the first episode.

### 2.4 Sampling Procedure

**Figure 1:**
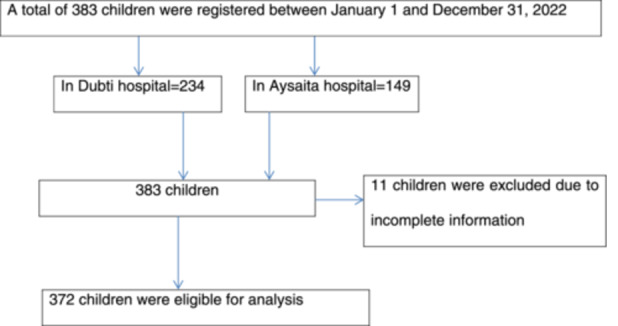
Diagrammatic flowchart for the selection of study subjects in public hospitals.

The study subjects were selected from Dubti and Aysaita hospitals situated in the Awsi Resu Zone in the Afar region. These hospitals were deliberately selected because of the availability of a sufficient number of severe acute malnutrition under‐five children, enabling us to obtain an adequate sample size. Charts and registration books from Dubti Hospital and Aysaita Hospital for 234 and 149 children with severe acute malnutrition aged 6–59 months, respectively, were retrospectively reviewed from March 1 to 20, 2023. Eleven children aged 6–59 months with incomplete information were excluded. Finally, 372 eligible children aged 6–59 months with severe acute malnutrition between the specified study periods were obtained. Those who met the eligibility requirements were reviewed one by one, and the data was entered into the pre‐structured data abstraction form (Figure 1).

### 2.5 Data Collection Tools and Procedures

Data were collected retrospectively from a stabilizing center for children aged 6–59 months with severe acute malnutrition from March 1 to 20, 2023. A data abstraction tool was prepared from the Ethiopian standard treatment protocol for the management of severe acute malnutrition in 2019 (FMOH 2019), a registration book, a medical history sheet, and severe acute malnutrition monitoring multi‐chart. The data was collected by four Bachelor of Science (BSc) nurses, two in each hospital, who have been working in the selected hospitals. There was one supervisor for each hospital. The data collectors and supervisors received 1 day of training on data extraction methods to ensure the quality of the data. Before the start of data collection, consistency between data recording systems and the prepared checklist was verified by randomly selecting and completing a few chart reviews, which led to minor amendments. Moreover, the supervisor and principal investigators provided strict follow‐up and supervision during the whole data collection period.

### 2.6 Variables and Operational Definitions

The dependent variable was the time to recover from severe acute malnutrition to normal nutritional status. The code for children recovered from severe acute malnutrition was 1, and the code for those who were censored was 0.

The independent variables are: socio‐demographic (age, sex, residence, admission type, and breastfeeding status), clinical features (malnutrition type, edema, fever, vomiting, diarrhea, loss of appetites, dehydration, hypothermia, hypoglycemia, convulsion, unconsciousness, lethargy, and eye signs of vitamin A deficiency), comorbidity variables (skin infection, TB, HIV, anemia, pneumonia, and malaria), and supporting and medication treatments.

Severe acute malnutrition (SAM) was defined as WHZ < −3 or MUAC < 115 mm, or the presence of bilateral pitting edema, or both (Lindsey Lenters and Bhutta 2016).

In this study, recovery was considered an event, while death, default, and nonresponse to treatment were considered censored. These terms were defined as follows in accordance with the Ethiopian national guidelines for the management of severe acute malnutrition (FMOH 2019):

Event (recovery): The dependent (outcome) variable of interest is time until an event occurs. Therefore, the current study defines a dependent variable of interest as the time‐to‐recovery of children from severe acute malnutrition to normal nutritional status. Children were recorded as event or recovered when they met the following criteria for discharge, which included being free of medical complications and edema, as well as gaining and maintaining a sufficient weight that was WFH and MUAC greater than or equal to 85% and 12.5 cm, respectively.

Death: A child dies while receiving treatment at the stabilizing center.

Defaulted: Absent for two consecutive visits.

Nonresponse to treatment: A patient who remained in treatment in the stabilizing center does not reach the severe acute malnutrition discharge criteria at the end of the follow‐up.

### 2.7 Data Management and Analysis

The data were coded, entered, and cleaned by Epi‐Info version 7 software and then exported to SPSS version 26. The missed variables and values were examined using frequencies and cross‐tabulations. In this study, survival analysis was used for data analysis. Survival analysis is simply time‐to‐event, in this case, time‐to‐recovery of children from severe acute malnutrition to normal nutritional status. Suppose that T is a random variable denoting the survival time of children with severe acute malnutrition. Therefore, T is defined as a waiting time until an event (recovery) occurs and is also regarded as a nonnegative continuous random variable with the probability density function (pdf) f(t) and cumulative distribution function (cdf) F(t), given by F(t) = P(T ≤ t), which provides the probability that an event (recovery) has occurred at time t. The survival function (S(t)) is defined as the probability that a child with severe acute malnutrition survives for longer than the specified time t. It is given by S(t) = P(T > t) = 1‐F(t), which is the complement of the cdf. Hence, S(t) gives the probability that the random variable T exceeds the specified time t. The hazard function of T, which is defined as an instantaneous potential per unit time for an event (recovery) to occur if the event has persisted up to time t, is denoted by h(t). Therefore, the hazard function is given by h(t) = f(t)/1‐F(t). This equation may also be expressed as h(t) = f(t)/S(t). The important feature of time‐to‐event data is censoring. Censoring occurs when there is information about a particular patient's survival time, but we do not know the exact survival time. Some children with severe acute malnutrition are censored (unrecovered, death, default, and nonresponse to treatment) because the recovery of children from severe acute malnutrition to normal nutritional status does not take place before the study ends. Survival function is very useful for comparing the survival progress of at least two groups. The Kaplan–Meier survival curve and the log‐rank test were used to assess whether the categorical variables had different recovery rates. Person time was calculated. In this study, person time was reported in child days. Total child days were calculated by adding the follow‐up times for each child from admission to the occurrence of an event or censors. The incidence rate was determined by dividing the total number of recovered children by the total number of child days. Bivariable and multivariable Cox regression models were used to determine factors that affected the recovery rate of children from severe acute malnutrition. Variables with *p* values of less than 0.05 in the bivariable analysis were considered eligible for the multivariate Cox proportional hazard model. A 95% confidence interval (CI) with the HR was calculated, and variables in the multivariate Cox proportional hazards model with *p* values less than 0.05 were considered significant predictors of the outcome variable. The basic assumptions of the Cox proportional hazard model were assessed by the Schoenfeld residual test (the global test) and a *p*‐value less than 0.10 indicates that the variable under investigation does not meet this assumption. The variance inflation factor (VIF) was used to check for multicollinearity. Finally, the results were presented in tables and figures by using frequency, percentages, and 95% CI with HR.

### 2.8 Ethical Issues

Ethical approval was obtained from a research and ethics review committee of the medical and health science college, Samara University, with an ethical approval code of SU/CMH/PG/112. Informed consent was waived by the ethical review committee of the College of Medical and Health Sciences, Samara University, due to the use of secondary data. Managers at Aysaita and Dubti Hospitals, as well as the person in charge of the health management information system, gave their informed consent before any personal cards were used for data collection. Participants in the study did not contact data collectors directly. Each patient's parent or legal guardian did not give permission for this study due to the use of secondary data. When data from the documents was collected, confidentiality and anonymity were respected. The data was not utilized for other purposes other than this research. Each patient card was coded, and the data collectors could only access the codes that the investigators had given them to identify each patient card.

## 3 Results

### 3.1 Socio‐Demographic Characteristics of Children

A total of 372 eligible children with severe acute malnutrition aged 6–59 months who were admitted between January 1 and December 31, 2022, were included. Most of the children (41.1%) were in the age range of 12–23 months. Female children represented more than half (51.6%). Rural children accounted for 53.5%. Newly admitted children were 75.8%, while 14% and 10.2% were readmitted and transferred in, respectively. Two‐thirds of children were breastfeeding (Table 2).

**Table 2:** Socio‐demographic characteristics of children.

Variables	Categories	Frequency	Percentages
Age (in months)	6–11	87	23.4
	12–23	153	41.1
	24–35	74	19.9
	36–47	20	5.4
	48–59	38	10.2
Sex	Males	180	48.4
	Females	192	51.6
Residence	Urban	173	46.5
	Rural	199	53.5
Admission type	New	282	75.8
	Readmission	52	14
	Transfer‐in	38	10.2
Breastfeeding	Yes	240	64.5
	No	132	35.5
Breastfeeding children	Under two years	183	76.2
	Two years and greater	57	23.8

### 3.2 Clinical Features and Comorbidities in Children

Half of the children (50.8%) had marasmus, and 57.9% had grade two edema. Children experiencing symptoms of fever, vomiting, and appetite loss were 49.7%, 87%, and 53.7%, respectively, while those experiencing diarrhea, dehydration, and hypothermia were 55.4%, 49.5%, and 43.8%, respectively. Thirty‐six percent, 44.3%, 36%, and 58.3% of children experienced convulsions, unconsciousness, and lethargy, respectively. Children who had pneumonia, tuberculosis, malaria and HIV represented 43.3%, 15.3%, 29%, and 10%, respectively (see more details in Table 3).

**Table 3:** Clinical features and comorbidities in children.

Variables	Categories	Event (recovery)		Censored (death, default, or nonresponse)		Total *N* (%)
		n	%	n	%	
Malnutrition type	Marasmus	108	29	81	21.8	189 (50.8)
	Marasmus‐kwashiorkor	71	19.1	51	13.7	122 (32.8)
	Kwashiorkor	37	9.9	24	6.5	61 (16.4)
Types of edema	Grade^+^	26	14.2	14	7.7	40 (21.9)
	Grade^++^	61	33.3	45	24.6	106 (57.9)
	Grade^+++^	21	11.5	16	8.7	37 (20.2)
Fever	Yes	107	28.7	78	21	185 (49.7)
	No	109	29.3	78	21	187 (50.3)
Appetites loss	Yes	201	54	122	33	323 (87)
	No	15	4	34	9	49 (13)
Vomiting	Yes	112	30	88	23.7	193 (53.7)
	No	104	28	68	18.3	179 (46.3)
Diarrhea	Yes	113	30.4	93	25	106 (55.4)
	No	103	27.7	63	16.9	166 (44.6)
Dehydration	Yes	96	25.8	88	23.7	184 (49.5)
	No	120	32.2	68	18.3	188 (50.5)
Hypothermia	Yes	98	26.3	65	17.5	163 (43.8)
	No	118	31.7	91	24.5	209 (56.2)
Hypoglycemia	Yes	155	41.7	116	31.2	271 (72.9)
	No	61	16.4	40	10.7	101 (27.1)
Convulsion	Yes	111	29.9	96	25.8	207 (55.7)
	No	105	28.2	60	16.1	165 (44.3)
Unconscious	Yes	70	18.8	64	17.2	134 (36)
	No	146	39	92	25	238 (64)
Lethargy	Yes	121	32.5	96	25.8	217 (58.3)
	No	95	25.6	60	16.1	155 (41.7)
Comorbidity	Yes	114	30.7	80	21.5	194 (52.2)
	No	102	27.4	76	20.4	178 (47.8)
Skin infection	Yes	73	19.6	50	13.4	123 (33)
	No	143	38.5	106	28.5	249 (67)
Children	With pneumonia	92	24.8	69	18.5	161 (43.3)
	Without pneumonia	124	33.3	87	23.4	211 (56.7)
Children	With TB	26	7	31	8.3	57 (15.3)
	Without TB	190	51.1	125	33.6	315 (84.7)
HIV	Positive	14	3.8	23	6.2	37 (10)
	Negative	202	54	133	36	335 (90)
Children	With anemia	69	18.6	44	11.8	113 (30.4)
	Without anemia	147	39.5	112	30.1	259 (69.6)
Children	With malaria	61	16.4	47	12.6	108 (29)
	Without malaria	155	41.7	109	29.3	264 (71)
Eye signs of vitamin A deficiency	Yes	31	8.3	25	6.7	56 (15)
	No	185	50	131	35	316 (85)

*Note:* +: one, ++: two, +++: three.

### 3.3 Routine Medication and Supporting Treatments for Children With Severe Acute Malnutrition

Thirty‐six percent of children were in a nasogastric tube, while 21.5% received blood transfusions. Children with Resomal resuscitation were 47.8% and 22.6% were in IV fluid. F‐75 and F‐100 were given to 96.7% and 76.6% of children, respectively. Routine antibiotics, antimalarial, and deworming were taken by 80.9%, 29%, and 40.9% of children, respectively. Children with RUTF were 72.6% (Table 4).

**Table 4:** Routine medication and supporting treatments for children with severe acute malnutrition.

Variables	Categories	Event (recovery)		Censored (death, default, or nonresponse)		Total *N* (%)
		n	%	n	%	
Nasogastric tube	Yes	70	18.8	64	17.2	134 (36)
	No	146	39.3	92	24.7	238 (64)
Blood transfusion	Yes	46	12.4	34	9.1	80 (21.5)
	No	170	45.7	122	32.8	292 (78.5)
ReSomal	Yes	92	24.7	86	23.1	178 (47.8)
	No	124	33.4	70	18.8	194 (52.2)
IV fluid	Yes	52	14	32	8.6	84 (22.6)
	No	164	44.1	124	33.3	288 (77.4)
F‐75	Yes	212	57	148	39.7	360 (96.7)
	No	4	1.1	8	2.2	12 (3.3)
F‐100	Yes	207	55.6	78	21	285 (76.6)
	No	9	2.4	78	21	87 (23.4)
Potassium permanganate	Yes	73	19.6	50	13.5	123 (33.1)
	No	143	38.4	106	28.5	249 (66.9)
Zinc supplement	Yes	108	29	77	20.7	185 (49.7)
	No	108	29	79	21.3	187 (50.3)
Vitamin A	Yes	85	22.9	54	14.5	139 (37.4)
	No	131	35.2	102	27.4	233 (62.6)
Folic acid	Yes	166	44.6	122	32.8	288 (77.4)
	No	50	13.4	34	9.2	84 (22.6)
Ready‐to‐use therapeutic food (RUTF)	Yes	168	45.2	102	27.4	270 (72.6)
	No	48	12.9	54	14.5	102 (27.4)
Routine antibiotic	Yes	180	48.4	121	32.5	301 (80.9)
	No	36	9.7	35	9.4	71 (19.1)
Antimalarial	Yes	61	16.4	47	12.6	108 (29)
	No	155	41.7	109	29.3	264 (71)
Deworming	Yes	91	24.5	61	16.4	152 (40.9)
	No	125	33.6	95	25.5	220 (59.1)
Measles vaccine	Yes	84	22.6	47	12.6	131 (35.2)
	No	132	35.5	109	29.3	241 (64.8)

### 3.4 Recovery Rates and Treatment Outcomes for Children With Severe Acute Malnutrition

The outcomes of treatments were that 58.1% of children recovered, 19.4% defaulted (absent for two consecutive visits), 14% died, and 8.6% had no response to treatment. Twenty‐eight days of the follow‐up period contributed to 4879 child days, with a median recovery time of 15.97 days. The overall incidence rate was 4.43 per 100 child days (95% CI: 3.88–5.04). There was a 25.0% probability of recovery between 6 and 9 days, a 63.0% probability at the end of the second week, an 83.0% probability at the end of the third week, and a 96.0% probability at the end of the fourth week. The density probability showed that the highest frequency of recovery rates occurred between 9 and 12 days. In accordance with the hazard rate, the risk of children failing to recover from severe acute malnutrition increased when hospital stays were prolonged (Table 5).

**Table 5:** Life table analysis for the recovery rate of children with severe acute malnutrition.

Interval start time in day	Number entering interval	Withdraw	Exposed to risks	Terminal events	Proportion terminating	Proportion of surviving at the end of interval	Cumulative proportion surviving	Probability density	Hazard rate
0–3	372	12	366	15	0.04	0.96	0.96	0.014	0.01
3–6	345	16	337	19	0.06	0.94	0.9	0.018	0.02
6–9	310	37	291.5	49	0.17	0.83	0.75	0.051	0.06
9–12	224	33	207.5	53	0.26	0.74	0.56	0.064	0.1
12–15	138	25	125.5	42	0.33	0.67	0.37	0.063	0.13
15–18	71	22	60	25	0.42	0.58	0.22	0.052	0.18
18–21	24	2	23	5	0.22	0.78	0.17	0.016	0.08
21–24	17	5	14.5	5	0.34	0.66	0.11	0.02	0.14
24–27	7	4	5	3	0.6	0.4	0.04	0.022	0.29

### 3.5 Comparing the Recovery Times of the Two Groups With Severe Acute Malnutrition Using the Kaplan–Meier Survival Function

Using the log‐rank test, the survival time between groups of various categorical predictors was determined. Children with and without TB had significantly differing survival times (*χ* ^2^ = 4.493, *p* = 0.034). The Kaplan–Meier (KM) survival curve for children without TB was steeper and had a shorter median length (15.6 days) than children with TB, with a longer median duration (18.2 days) (Figure S1). There was a significant variation in the median survival time between HIV‐positive and HIV‐negative children (*χ* ^2^ = 10.3, *p* = 0.001). HIV‐negative children recovered from severe acute malnutrition more quickly and in a shorter survival median time (15.5 days) than HIV‐positive children, who recovered more slowly and in a longer survival median time (19.48 days) (Figure S2). Children without dehydration (Figure S3), unconsciousness (Figure S4), convulsion (Figure S5), and lethargy (Figure S6) also showed survival curves that were significantly steeper than those of children with these related conditions (*p* < 0.05). Children who experienced dehydration (17.3 days), convulsions (17 days), unconsciousness (17 days), and lethargy (16.9 days) experienced longer median survival times than their counterparts who did not experience dehydration (14.6 days), convulsions (15.2 days), unconsciousness (15.2 days), or lethargy (14.6 days), respectively. However, severe acute malnutrition children with pneumonia (*χ* ^2^ = 0.48, *p* = 0.49), diarrhea (*χ* ^2^ = 0.84, *p* = 0.359), anemia (*χ* ^2^ = 2.56, *p* = 0.11), malaria (*χ* ^2^ = 1.45, *p* = 0.228), hypoglycemia (*χ* ^2^ = 0.145, *p* = 0.703), and hypothermia (*χ* ^2^ = 3.42, *p* = 0.064) did not reveal significant differences in their survival curve with their counterparts who did not have these conditions.

### 3.6 Predictors of the Recovery Time of Children Aged 6–59 Months With Severe Acute Malnutrition

The results of the global test showed that the assumption was violated (*p* = 0.007). This indicated that there is statistical evidence that at least one variable violates the proportional hazards assumption or the hazard ratio is not constant over time. Hence, a time‐varying covariate Cox proportional hazard model was used. Variables with *p*‐values less than 0.05 in the binary cox regression were included in the multivariable cox regression. The multivariate cox regression revealed that children who were HIV‐negative and those who did not get IV antibiotics recovered from severe acute malnutrition more quickly than those who were HIV‐positive and received IV antibiotics, respectively. On the other hand, children who lived in rural areas and did not take F‐100 supplements recovered more slowly than children who took F‐100 supplements and lived in urban areas.

Children living in rural areas had a 30% lower recovery rate from severe acute malnutrition than children living in urban areas (AHR = 0.7, 95% CI: 0.5–0.94). Children without IV antibiotics had a 1.4 times higher recovery rate than those with IV antibiotics (AHR = 1.4, 95% CI: 1.03–2.0). Children who did not take F‐100 (AHR = 0.85, 95% CI: 0.79–0.91) were 15% less likely to recover from severe acute malnutrition than those who did. HIV‐negative children were 76% more likely to recover from severe acute malnutrition than HIV‐positive children (AHR = 1.76, 95% CI: 1.1–3.3) (Table 6).

**Table 6:** Predictors of severe acute malnutrition in under five children.

Variables	Categories	Recovered		Censored		CHR (95% CI)	AHR
		*n*	%	*n*	%		
Residence	Urban	112	30.1	61	16.4	1	1
	Rural	104	28	95	25.5	0.65 (0.5–0.85)	0.7 (0.5–0.94)*
Nasogastric intubation	Yes	70	18.8	64	17.2	1	1
	No	146	39.3	92	24.7	1.3 (1.0–1.8)	1.3 (0.97–1.7)
Zink supplement	Yes	108	29	77	20.7	1	1
	No	108	29	79	21.3	1.3 (1.01–1.7)	1.2 (0.9–1.6)
F‐100	Yes	207	55.6	78	21	1	1
	No	9	2.4	78	21	0.17 (0.09–0.32)	0.85 (0.79–0.91)*
Potassium permanganate	Yes	73	19.6	50	13.5	1	1
	No	143	38.4	106	28.5	0.7 (0.5–0.9)	0.99 (0.91–1.01)
Iv antibiotics	Yes	165	44.4	124	33.3	1	1
	No	51	13.7	32	8.6	1.6 (1.1–2.1)	1.4 (1.03–2.0)*
Appetite's loss	Yes	201	54	122	33	1	1
	No	15	4	34	9	0.49 (0.29–0.8)	0.78 (0.45–1.34)
TB	Yes	26	7	31	8.3	1	1
	No	190	51.1	125	33.6	1.5 (1.01–2.3)	0.88 (0.54–1.44)
HIV	Yes	14	3.8	23	6.2	1	1
	No	202	54	133	36	2.3 (1.3–3.9)	1.76 (1.1–3.3)*
Dehydration	Yes	96	25.8	88	23.7	1	1
	No	120	32.2	68	18.3	1.6 (1.2–2.1)	1.86 (0.88–3.9)
Convulsion	Yes	111	29.9	96	25.8	1	1
	No	105	28.2	60	16.1	1.4 (1.14–1.9)	0.95 (0.5–1.67)
Unconscious	Yes	70	18.8	64	17.2	1	1
	No	146	39	92	25	1.3 (1.03–1.8)	0.7 (0.45–1.2)
Lethargy	Yes	121	32.5	96	25.8	1	1
	No	95	25.6	60	16.1	1.5 (1.2–2.0)	1.0 (0.6–1.48)

* Statistically significant.

## 4 Discussion

The objective of the study was to determine the time to recovery from severe acute malnutrition and its predictors in the pastoral region of Afar, Ethiopia. The study is essential to improve the recovery rate of under‐five children with severe acute malnutrition and reduce the mortality rate due to malnutrition, particularly in the recurrently drought‐affected regions of Afar, Ethiopia, where the burden of the disease is high (Gebre et al. 2019). The study was also useful to improve the service management of children with severe acute malnutrition.

The median recovery time in this study was 15.97 days, with minimum and maximum recovery times of 3 and 29 days, respectively. This contributed to a total of 4879 children per day, with a recovery incidence rate of 4.43 per 100 children per day, which was lower than the earlier district studies in Ethiopia (Adimasu et al. 2020; F. K. Bizuneh et al. 2022; Kidane et al. 2023; Wondim et al. 2020). The current study's recovery incidence rate was higher than the studies conducted in the Oromia region (Eyi et al. 2022), North‐West Ethiopia (Mamo et al. 2019), and Southern Ethiopia (Fikrie et al. 2019). However, the present study's recovery rate of 4.43 per 100 children per day was consistent with a previous study conducted in the Jimma district in Ethiopia that reported a rate of 4.06 per 100 children per day (Hussen Kabthymer et al. 2020). Numerous factors, including variations in socio‐demographic characteristics, economic factors, environmental factors, clinical factors, and improper implementations of treatment protocols (Bitew, Alebel, Worku 2020), could be the cause of these discrepancies. Most of the previous studies were conducted at least 5 years ago, so the distinct study period could be the reason for this disparity.

The median time to recover from severe acute malnutrition in our study was 15.97 days. The Sphere standard recommended that the recovery time of children admitted to stabilization centers should be less than 1 month (Sphere 2018). Literature in Ethiopia showed that the median recovery time varied from 14 days (Wondim et al. 2020) to 8.7 weeks (Atnafe et al. 2019) in Ethiopia. The median recovery time of the current study was significantly faster than the previous studies conducted in Ethiopia (Atnafe et al. 2019; Eyi et al. 2022; Fikrie et al. 2019; Hussen Kabthymer et al. 2020; Mamo et al. 2019; Simachew et al. 2020; Wondie et al. 2022); however, it was slower than another previous study in Ethiopia (Wondim et al. 2020). The current median recovery time was nearly comparable to earlier studies conducted in Ethiopia (Adimasu et al. 2020; Baraki et al. 2020; F. K. Bizuneh et al. 2022; Kidane et al. 2023). Discrepancies may be due to inequalities in health care service standards, differences in the quantity and quality of healthcare providers (Addis and Gebeyehu Wondmeneh 2023), a lack of adequate treatment supply, variations in follow‐up time, and variations in the occurrence of comorbidities. The late detection of severe acute malnutrition and the late referral system to the stabilization centers could also contribute to variations in the recovery time. The Afar region is among the emerging pastoralist regions in Ethiopia, where infrastructure and services are unequally distributed (Desalegn and Solomon 2022). Slow recovery in the Afar region may be due to the fact that the health services are inadequate, poorly equipped, hard to access, and incompatible with the livelihood of the pastoral community's system (Abdulkadr 2021).

At the end of the follow‐up period, 58.1% of children recovered, 19.4% defaulted, 14% died, and 8.65% did not respond to treatments. This is below the Sphere minimum standard (Sphere 2018) and the national (FMOH 2019) minimum acceptable cure rate, which stated that the recovery rate was > 75%, defaulted < 15%, and died < 10%. The discrepancy can result from the sphere standards being mostly ideal and consensus‐based rather than evidence‐based. The lack of contextualization and stakeholder input in standards could be another cause. This magnitude was also lower than those of studies conducted in sub‐Saharan Africa, which had 71.2% (Desyibelew et al. 2020). Moreover, it was lower than studies conducted at the national level that found that 70% and 72% (Bitew, Alebel, Worku 2020; Yazew et al. 2019) and district‐level studies of Ethiopia (Adimasu et al. 2020; Atnafe et al. 2019; Baraki et al. 2020; F. K. Bizuneh et al. 2022; Eyi et al. 2022; Fikrie et al. 2019; Hussen Kabthymer et al. 2020; Kidane et al. 2023; Mamo et al. 2019; Simachew et al. 2020; Wondim et al. 2020). The reason for this low recovery rate could be a high default rate in the current study. Another possible explanation for this low recovery rate may be the study area's 3‐year history of conflict or war (FAO 2021) and recurrent drought (Wondmeneh 2022) contributing to food insecurity; the study area's inadequate water supply (Libey et al. 2022) and the presence of teenage mothers due to early marriage (Alem et al. 2020; Dessalegn et al. 2020) could all have an effect on recovery rates. However, the percentage (58.1%) of the current study was somewhat higher than that of another previous study in southern Ethiopia (54.4%) (Wondie et al. 2022).

Children without TB and those with HIV‐free status had faster recovery times when compared to children with TB and HIV infections, respectively. This might be because children with HIV plus severe acute malnutrition had a higher level of inflammatory mediators than children with severe acute malnutrition alone (Sturgeon et al. 2023). Children with dehydration, unconscious and lethargy had longer recovery times than their counterparts. The explanation could be that dehydration disturbs cell metabolism, causing cell breakdown (Saghaleini et al. 2018) that may lead to longer recovery compared to non‐dehydrated children. Significant predictors of time to recovery from severe acute malnutrition were living in rural areas, a lack of F‐100 supplements, being without IV antibiotics, and being HIV‐free. Children who lived in rural areas had a 30% lower recovery rate from severe acute malnutrition than those who lived in urban areas. The possible explanation may be that rural under‐five children were experiencing more severe undernutrition than urban children (Anik et al. 2021). The other might be the occurrence of poor sanitation, such as the absence of WASH packages in rural households compared to those in urban households (Altmann et al. 2018). Children who did not receive the F‐100 milk supplement were 15% less likely to recover than those who did receive it. The present evidence aligns with the findings of earlier studies, which indicated that children who took F‐100 recovered more quickly than those who did not (F. Bizuneh et al. 2021; Fikrie et al. 2019). The high recovery could be attributed to the fact that F‐100 contained high‐energy products with rich nutrient content. Children without IV antibiotics had a 1.4 times higher recovery rate than those with IV antibiotics, which could be that those children with IV antibiotics were severely complicated, leading to a delayed recovery (Tidjani Alou et al. 2017). This finding is consistent with the previous findings that found amoxicillin provided better recovery (Husen et al. 2022; Hussen Kabthymer et al. 2020). Children with HIV‐negative children recovered from severe acute malnutrition 76% more than children with HIV‐positive children. This evidence is in line with the previous studies that reported that children with severe acute malnutrition who had HIV had poor recovery rates compared to non‐HIV‐infected children with severe acute malnutrition (Aye et al. 2023; Baraki et al. 2020; Oumer et al. 2021). HIV infection, especially prolonged infection exposure, weakens the immune system and eventually wears out immune cells (Korencak et al. 2019; Sainz et al. 2022). Immune dysfunction can be directly related to pathological malnutrition processes such as malabsorption, elevated metabolic demand, dysfunction of the growth hormone, and increased susceptibility to infection (Bourke et al. 2016).

The first drawback of this study was its retrospective design, which depended on only records and excluded a few patient records with incomplete data from the analysis, possibly underestimating or biasing if the excluded data were relevant to the recovery rate. Furthermore, because this study was facility‐based, it did not include children with severe acute malnutrition at the community level. But this study's strength is that it's one of the few studies in Ethiopia, especially in the context of the Afar region that investigated treatment outcomes and factors affecting recovery time for 6‐ to 59‐month‐old children with severe acute malnutrition. This information is essential to raising public health officials' awareness and directly enhancing the management of severe acute malnutrition in the health facilities in the Afar region. The results of this study were also used to advance the fight against hunger, help achieve the 2030 target of ending all types of malnutrition(UN 2023), and address the nutritional needs of children.

## 5 Conclusion

In this study, although the median recovery time from severe acute malnutrition was in the acceptable range of the standard sphere and the national guideline, the recovery rate was below the minimum acceptable cutoff point. The death and default rates also exceeded the minimum acceptable range. Children who consume F‐100 can recover quickly from severe acute malnutrition; thus, it was essential to enhance the F‐100 supplement and scale up its expansion at the stabilization center. Special attention should be given to children with HIV infection, IV antibiotic use, and living in rural areas. Severe acute malnutrition should be identified promptly and urgently referred to a stabilization center to reduce the risk of malnutrition complications, increase recovery rates, and save lives.

## Author Contributions

Temesgen Gebeyehu Wondmeneh and Amarech Giruma wrote the original draft, developed the methodology, under project supervision, analyzed the data, distributed resources, and wrote the main manuscript text. Both authors reviewed the manuscript and agreed to submit it.

## Consent

The authors have nothing to report.

## Conflicts of Interest

The authors declare no conflicts of interest.

## Supporting information

Supporting information.

Supporting information.

Supporting information.

Supporting information.

Supporting information.

Supporting information.

## Data Availability

The datasets used to support the findings of this study are available from the corresponding author upon request.
