# Supplementary figures and images for: Time to Recovery From Severe Acute Malnutrition to Normal Nutritional Status and Its Predictors Among Children Aged 6–59 Months in North‐East Ethiopia
Source: Matern Child Nutr. 2025 Feb 16;21(3):e13808. doi: 10.1111/mcn.13808 (PMC12150153; doi:10.1111/mcn.13808)

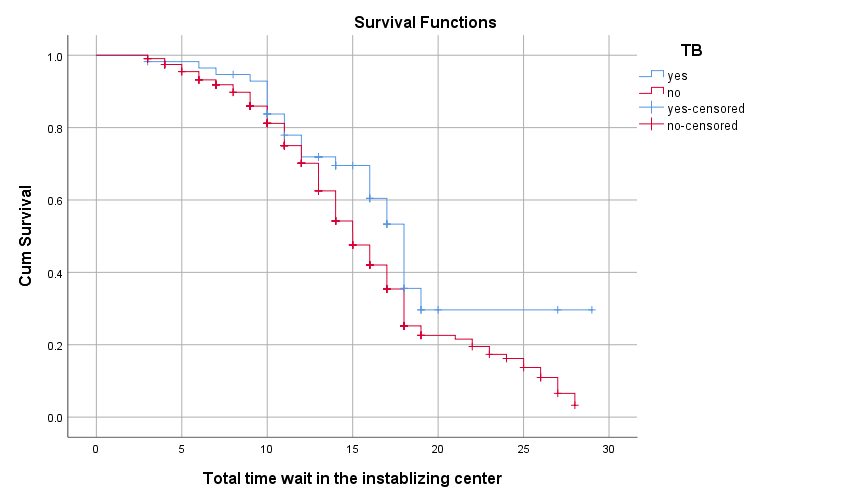


S Fig.1 Comparing children with and without TB

Supplement: Supplementary file 1 — Supporting information. [file MCN-21-e13808-s005.docx]

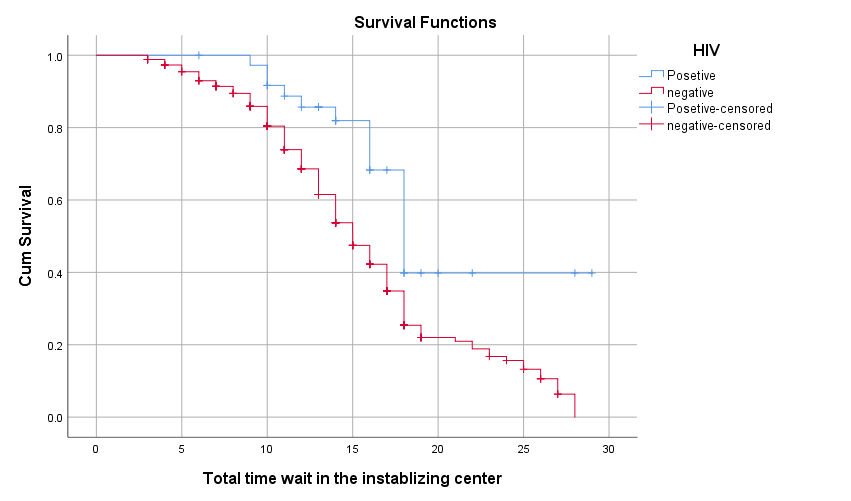


S Fig.2 Comparing HIV-infected children with HIV-free children

Supplement: Supplementary file 2 — Supporting information. [file MCN-21-e13808-s006.docx]

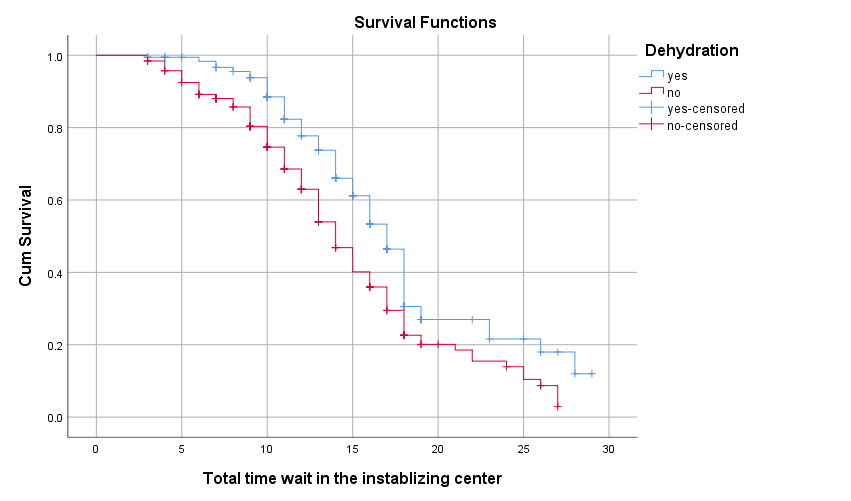


S Fig.3 A comparison of children with and without dehydration

Supplement: Supplementary file 3 — Supporting information. [file MCN-21-e13808-s002.docx]

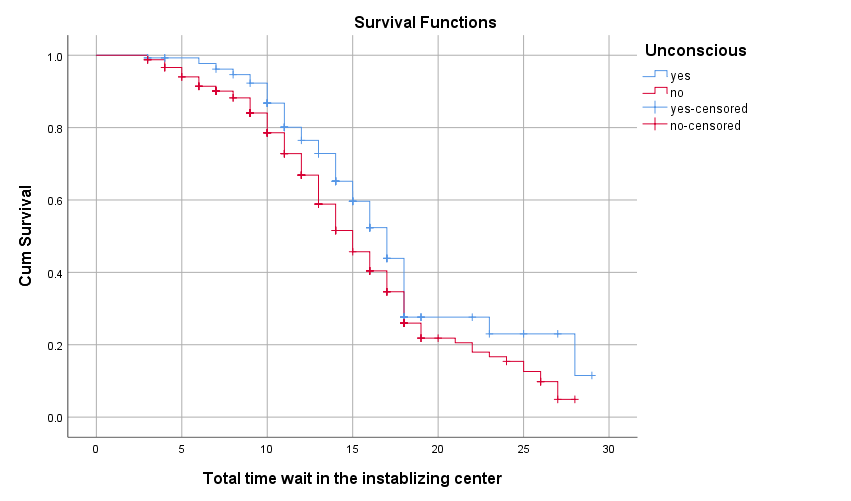


S Fig.4 A comparison of children with and without consciousness

Supplement: Supplementary file 4 — Supporting information. [file MCN-21-e13808-s001.docx]

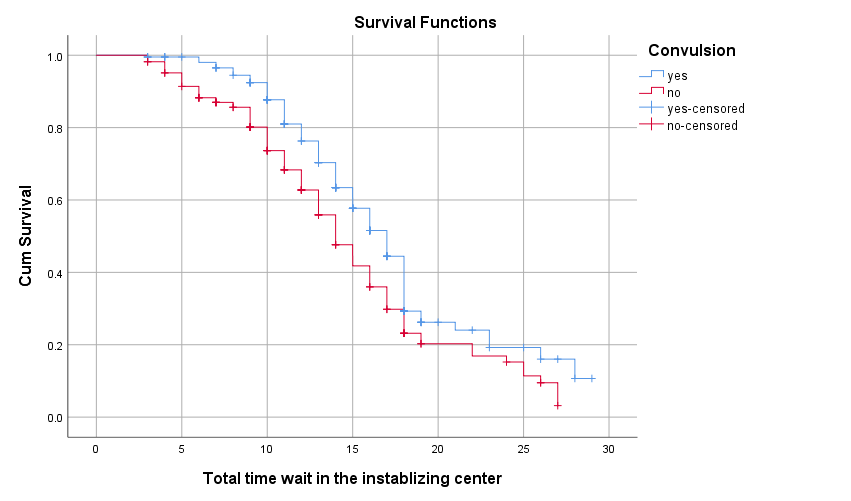


S Fig.5 Comparison of children with and without convulsions

Supplement: Supplementary file 5 — Supporting information. [file MCN-21-e13808-s003.docx]

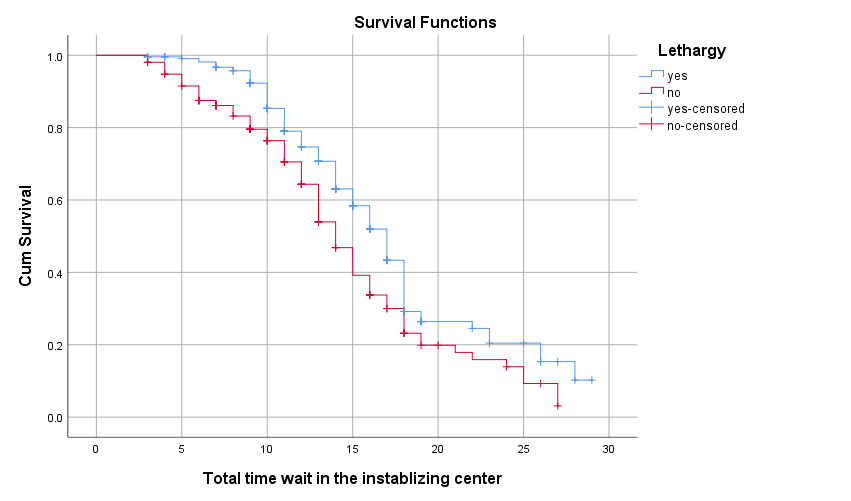
S Fig.6 A comparison of lethargic children with non-lethargic children

Supplement: Supplementary file 6 — Supporting information. [file MCN-21-e13808-s004.docx]
